# Development of a multicomponent vaccine for *Streptococcus pyogenes* based on the antigenic targets of IVIG

**DOI:** 10.1016/j.jinf.2016.02.002

**Published:** 2016-04

**Authors:** Mark Reglinski, Nicola N. Lynskey, Yoon Jung Choi, Robert J. Edwards, Shiranee Sriskandan

**Affiliations:** Faculty of Medicine, Imperial College London, Hammersmith Campus, Du Cane Road, London, W12 0NN, United Kingdom

**Keywords:** *Streptococcus pyogenes*, Group A *Streptococcus*, Vaccine, IVIG, Population immunity

## Abstract

**Objectives:**

Despite over a century of research and the careful scrutiny of many promising targets, there is currently no vaccine available for the prevention of *Streptococcus pyogenes* infection. Through analysis of the protective, anti-streptococcal components of pooled human immunoglobulin, we previously identified ten highly conserved and invariant *S. pyogenes* antigens that contribute to anti-streptococcal immunity in the adult population. We sought to emulate population immunity to *S. pyogenes* through a process of active vaccination, using the antigens targeted by pooled human immunoglobulin.

**Methods:**

Seven targets were produced recombinantly and mixed to form a multicomponent vaccine (Spy7). Vaccinated mice were challenged with *S. pyogenes* isolates representing four globally relevant serotypes (M1, M3, M12 and M89) using an established model of invasive disease.

**Results:**

Vaccination with Spy7 stimulated the production of anti-streptococcal antibodies, and limited systemic dissemination of M1 and M3 *S. pyogenes* from an intramuscular infection focus. Vaccination additionally attenuated disease severity due to M1 *S. pyogenes* as evidenced by reduction in weight loss, and modulated cytokine release.

**Conclusion:**

Spy7 vaccination successfully stimulated the generation of protective anti-streptococcal immunity *in vivo*. Identification of reactive antigens using pooled human immunoglobulin may represent a novel route to vaccine discovery for extracellular bacteria.

## Highlights

• Targets of population level immunity to *Streptococcus pyogenes* can be identified using pooled IVIG.
• Seven conserved targets were combined to form a new vaccine (Spy7).
• Vaccine antigen components were individually immunogenic.
• Vaccination with Spy7 reduced dissemination of invasive M1 and M3 *S. pyogenes*.
• The immuno-proteomic approach to vaccine discovery may be applicable to other pathogens.

## Introduction

*Streptococcus pyogenes* is often overlooked as a major cause of human disease owing to the rarity of severe *S. pyogenes* infection in the developed world. While the attendant morbidity and mortality of invasive syndromes such as necrotizing fasciitis and toxic shock are considerable, it is the rheumatogenic sequelae of non-invasive *S. pyogenes* infections that represent the most pressing global health burden. Such sequelae account for the majority of the >500,000 deaths per year worldwide attributed to streptococcal infection.^1^ Much of the burden of rheumatic heart disease (RHD) exists in developing countries where poverty and limited access to prompt antibiotic treatment may contribute to the development of autoimmunity.^2^ Although *S. pyogenes* remains exquisitely sensitive to beta-lactam antibiotics, the development of an effective vaccination is widely regarded as the most reliable way to reduce the global *S. pyogenes* disease burden.

Previous attempts to develop *S. pyogenes* vaccines involved the use of single antigen formulations containing well characterized virulence factors such as the M protein,^3^, ^4^ C5a peptidase^5^ and, more recently, SpyCEP.^6^, ^7^ However, there has been a recent shift towards the development of multicomponent vaccines for pathogenic bacteria, with the expectation that the inclusion of multiple targets will ensure longevity of efficacy and coverage. Several approaches to *S. pyogenes* vaccine antigen identification have been described including, classical reverse vaccinology,^7^, ^8^ serological identification of reactive surface antigens^9^ and antigenome technology.^10^ One study used three such approaches in conjunction with murine infection experiments to identify six highly conserved protective antigens, three of which (SpyCEP, streptolysin O and SpyAD) were developed commercially as a multicomponent vaccine.^11^ With the exception of the M protein, no vaccine candidates have reached clinical trials to date.

We have characterized the streptococcal surface protein targets of pooled human immunoglobulin (intravenous immunoglobulin G, IVIG) using twenty different *S. pyogenes* isolates representing four major *emm*/M types.^12^ IVIG is a clinical blood product that is purified from the blood of over one thousand healthy donors and therefore represents a useful surrogate of population level immunity. Among the identified *S. pyogenes* antigens, was a set of ten highly conserved, invariant surface proteins that were conserved across not only the 20 isolates tested, but also all genome-sequenced strains (Table 1). Several of these proteins had previously been evaluated as vaccine antigens using established *S. pyogenes* infection models.^5^, ^7^, ^10^, ^11^, ^13^ Given the ability of human immunoglobulin to protect against *S. pyogenes* infection, we hypothesized that a multicomponent vaccine containing these conserved invariant surface proteins would provide serotype independent protection against *S. pyogenes* infection. Here seven of the conserved, invariant targets of pooled human immunoglobulin were produced recombinantly and combined to form a novel multivalent vaccine that was evaluated in an experimental model of *S. pyogenes* infection. The data suggest that vaccination with these seven antigens may be sufficient to emulate the anti-streptococcal activity of pooled human immunoglobulin.

## Materials and methods

### Bacterial strains and growth conditions

*The S. pyogenes* isolates used in this study are listed in Table 1 and were routinely cultured from frozen stocks on Columbia horse blood agar or in Todd-Hewitt broth at 37 °C in 5% CO_2_. Strains were selected to represent four clinically relevant serotypes circulating in Europe and North America (Table 1). CovRS status of isolates was determined by sequence analysis as previously described.^14^ SpeB production was assessed by immunoblot analysis using a 1:1000 dilution of rabbit anti-SpeB antibody (Toxin Technology) and a 1:80,000 dilution of HRP-conjugated goat anti-rabbit IgG under standard conditions. *Escherichia coli* isolates were routinely cultured at 37 °C in LB broth or agar supplemented with 100 μg/ml of ampicillin. Liquid *E. coli* cultures were grown with agitation at 225 rpm.

### Selection of vaccine antigens

Eight of the ten highly conserved and invariant streptococcal surface antigens previously shown to be recognized by IVIG were cloned and expressed in *E. coli*.^12^ Two proteins were excluded; one on the grounds of its predicted cytoplasmic localization (chaperone protein DnaK), and the other owing to the significant degree of sequence identity to another antigen (nucleoside-binding protein). One of the antigens proved recalcitrant to expression using the methods applied (maltose/maltodextrin-binding protein). The seven resulting recombinant antigens were combined in equal amounts (10 μg/protein) to form a novel multicomponent vaccine denoted Spy7.

### Recombinant protein production

BamHI or NdeI-ended coding sequences for the selected antigens were amplified from *S. pyogenes* gDNA (strain H305) and cloned into pET-19b excluding any signal sequence and cell wall anchor domain (Supplementary Table 1). For *pulA* (*Spy_1972*) a 1.6 kb N-terminal fragment was amplified by PCR and cloned into the TA cloning vector PCR 2.1 (Invitrogen) to facilitate NdeI digestion. For *scpA*, a premature stop codon was inserted at amino acid 720 by site directed mutagenesis (QuikChange II XL, Agilent) to prevent removal of the N-terminal propeptide.^15^ Antigens were produced by IPTG-induced overexpression in One Shot^®^ BL21 (DE3) Chemically Competent *E. coli* (Invitrogen) according to the manufacturer's instructions. The induced BL21 cells were lysed with BugBuster (Novagen) and proteins were purified using the Ni-NTA purification system (Invitrogen) according to the manufacturer's instructions.

### Active immunization and cytokine analysis

Age matched (5–6 week old) female FVB/N mice (Charles River Laboratories International) housed in cages of six were vaccinated intramuscularly (left flank) with 70 μg of protein, emulsified 1:1 with Freund's complete adjuvant. Booster immunizations were given at 21 (right flank) and 35 days (left flank) in Freund's incomplete adjuvant. Controls were immunized with PBS and adjuvant alone. Tail bleeds were performed on day 41 (one day prior to infection) to confirm seroconversion. Mice were challenged intramuscularly (right flank) with ∼1 × 10^7^ CFU of *S. pyogenes* serotypes: M1, (strain H305); M3, (strain H330); M12, (strain H529) and M89, (strain H293) on day 42. At 24 h post infection the mice were weighed and culled, and blood was removed by cardiac puncture. The infected thigh, draining inguinal lymph node, spleen and liver were dissected, weighed and homogenized. Bacterial loads were quantified by plating. Challenge experiments were either conducted as a single study involving 12 mice per group (M1 and M3 challenges) or as two studies involving 6 mice per group (12 mice per group total, M12 and M89 challenges). Plasma cytokines in blood from infected mice were quantified using a mouse cytokine 20-plex panel (Invitrogen) according to the manufacturer's instructions.

### Seroconversion ELISAs

Plates were coated with 50 μl/well of either whole *S. pyogenes* cells (resuspended to an A_600_ of 0.5 in PBS) or 1 μg/well of recombinant protein and probed with plasma recovered on day 41 post-vaccination (prior to infection challenge). Bound antibodies were detected using 1:2000-diluted HRP-conjugated goat anti-mouse IgG (Sigma–Aldrich) and 50 μl/well tetramethylbenzidine (Sigma–Aldrich). The reaction was stopped through addition of 50 μl of 1 M H_2_SO_4_ and absorbance at 450 nm was measured. To determine the development and durability of the human antibody response to Spy7 antigens, ELISA plates were coated with 1 μg/well of recombinant protein and probed with 10-fold dilutions (1:1000–1:1,000,000) of acute and convalescent serum from a patient who had recovered from an *S. pyogenes* bacteraemia 8 weeks earlier, and who had not received IVIG. Bound antibodies were detected using a 1:2000 dilution of HRP-conjugated goat anti-human IgG. Normal human serum from a healthy adult was included as a control.

### Lymphocyte proliferation assays

For lymphocyte proliferation assays, female FVBn mice were vaccinated with a single 70 μg dose of Spy7 (n = 3) or sham vaccinated with PBS and adjuvant alone (n = 1). Draining inguinal lymph nodes were harvested at 12 d and disaggregated into single cell suspensions using a 70 μm cell sieve into RPMI (supplemented with 10% FBS, 2 mM l-glutamine and 500 μg/ml penicillin–streptomycin). 2 × 10^5^ cells/well were co-incubated in triplicate with 1 μg/well of antigen, or 28 μg/well of Spy7, in a final volume of 200 μl at 37 °C for 48 h. Cellular proliferation was measured using a colourimetric BrdU assay (Roche) according to the manufacturer's instructions. Absorbances generated by cells from Spy7 vaccinated animals were corrected for non-specific reactivity by subtraction of the mean absorbance generated by cells from the sham vaccination experiment.

### Ethics

All animal procedures were approved by the local ethical review process at Imperial College London and conducted in accordance with the relevant, UK Home Office approved, project licence. The collection and use of anonymized samples from patients with infection was approved by the relevant research ethics committee (NRES 06/Q0406/20).

### Statistical analysis

Data were analysed using GraphPad Prism (GraphPad Software Inc.) and are displayed as median and range, or mean and standard deviation. Statistical significance was accepted where *p < 0.05 using a two-tailed Mann–Whitney *U* (two groups) or Kruskal–Wallis one-way ANOVA with Dunn's post-test (three groups).

## Results

### Vaccination with Spy7 generates anti-streptococcal antibodies

**Figure 1:**
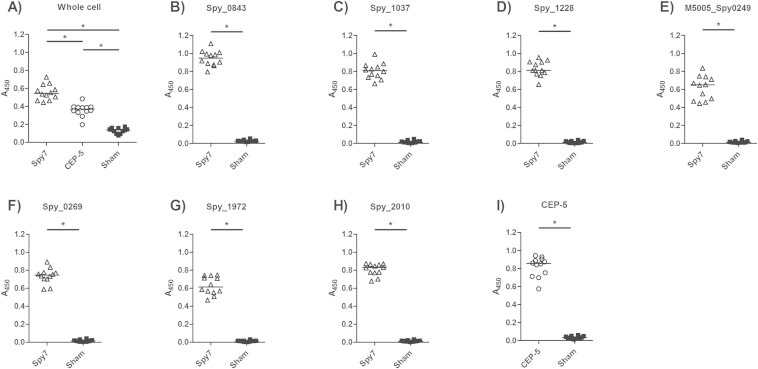
**Vaccination with Spy7 stimulates the generation of anti-streptococcal antibodies**. Plates were coated with (A) live M1 *S. pyogenes* (strain H305) or (B–I) 1 μg/well of recombinant protein and probed with a 1:8000 dilution of murine plasma recovered from Spy7 vaccinated (open triangles), CEP-5 vaccinated (open circles) or sham vaccinated (closed squares) mice. Bound antibodies were detected using a 1:2000 dilution of HRP-conjugated goat anti-mouse IgG. A) *p < 0.05 Kruskal–Wallis (Dunns post-test) B–I) *p < 0.05 two-tailed Mann–Whitney *U*.

To determine the efficacy of Spy7, female FVB/N mice were vaccinated with Spy7 or sham vaccinated with PBS and adjuvant alone. A third group was vaccinated with the SpyCEP fragment CEP-5 that has previously been shown to provide protection against experimental *S. pyogenes* infection.^6^, ^16^ 41 d following the start of the vaccination protocol, the anti-streptococcal activity of plasma from Spy7-vaccinated and sham-vaccinated mice was compared by whole bacterial cell ELISA (Fig. 1A). Comparison of the Spy7 and CEP-5 vaccinated plasma revealed that Spy7 generated a stronger anti-streptococcal antibody response than CEP-5 alone, most likely resulting from the inclusion of multiple surface protein targets in the Spy7 formulation. Each of the seven recombinant vaccine components also reacted with plasma from Spy7-vaccinated mice; indicating that each antigen had stimulated the humoural immune response, and thus was potentially contributing to the anti-streptococcal antibody response elicited by Spy7 (Fig. 1B–H). These levels of reactivity were comparable with the anti-CEP-5 reactivity measured in the mice vaccinated with CEP-5 alone indicating that the levels of circulating antibody generated by Spy7 vaccination may not necessarily be improved by increasing the concentration of the individual antigens (Fig. 1I).

The development and durability of the human antibody response to the Spy7 components was examined by ELISA, using acute and convalescent serum from a patient who had recovered from an invasive M1 *S. pyogenes* bacteraemia, and normal human serum from a healthy adult (Supplementary Fig. 1). In contrast to the acute sample (obtained on the day of presentation), convalescent serum (obtained 8 weeks after presentation) reacted strongly against six of the seven Spy7 components. The seventh component (hypothetical membrane associated protein, Spy_1037), displayed only modest reactivity. C5a peptidase (Spy_2010) and SpyAD (Spy_0269) were also shown to react strongly with the normal human serum sample, suggesting that once primed, the humoural immune response to these antigens may persist in circulation for extended periods.

### Vaccination with Spy7 prevents dissemination of serotype M1 and M3 *S. pyogenes*

**Figure 2:**
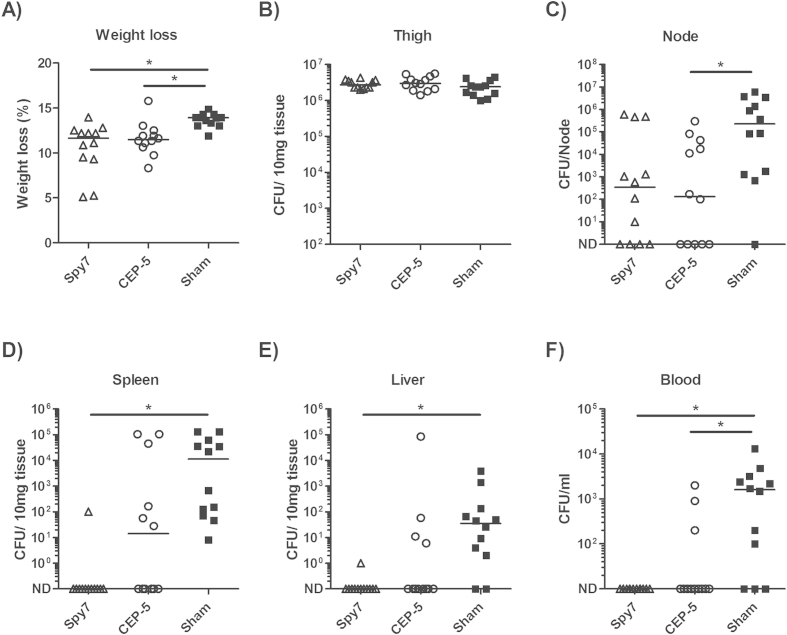
**Spy7 prevents dissemination of M1 *S. pyogenes* from an intramuscular focus of infection**. Three groups of twelve age-matched female FVB/N mice were vaccinated with Spy7 (open triangles) or CEP-5 (open circles), or sham vaccination with PBS and adjuvant alone (closed squares) and challenged with ∼1 × 10^7^ CFU/mouse of M1 *S. pyogenes* (strain H305). Solid lines indicate the median CFU recovered from each organ 24 h post infection. *p < 0.05 Kruskal–Wallis (Dunns post-test) ND: Not Detected.

Having established that Spy7 and CEP-5 had both elicited a strong humoural immune response, the vaccinated mice were challenged intramuscularly with 1 × 10^7^ CFU of live M1 *S. pyogene*s and the ability of each vaccine to prevent bacterial dissemination was assessed in comparison with sham vaccination. Percentage weight loss was lower in both the Spy7 and CEP-5 vaccinated mice compared to the sham vaccinated group, consistent with reduced illness severity (Fig. 2A). The bacterial burden in the infected thigh was comparable between groups (Fig. 2B). While a trend to reduction in bacterial burden in the inguinal lymph node was apparent in both groups, a statistically significant difference was only recorded between the CEP-5 vaccinated and sham vaccinated groups (Fig. 2C). Vaccine-induced immunity was sufficient to inhibit systemic bacterial dissemination to the spleen (Fig. 2D), liver (Fig. 2E), and blood (Fig. 2F) of the Spy7-vaccinated mice compared with the sham-vaccinated group. In contrast, no significant reduction in bacterial burden was reported in the spleen or liver of the CEP-5 vaccinated mice, when compared to the sham vaccinated group.

**Figure 3:**
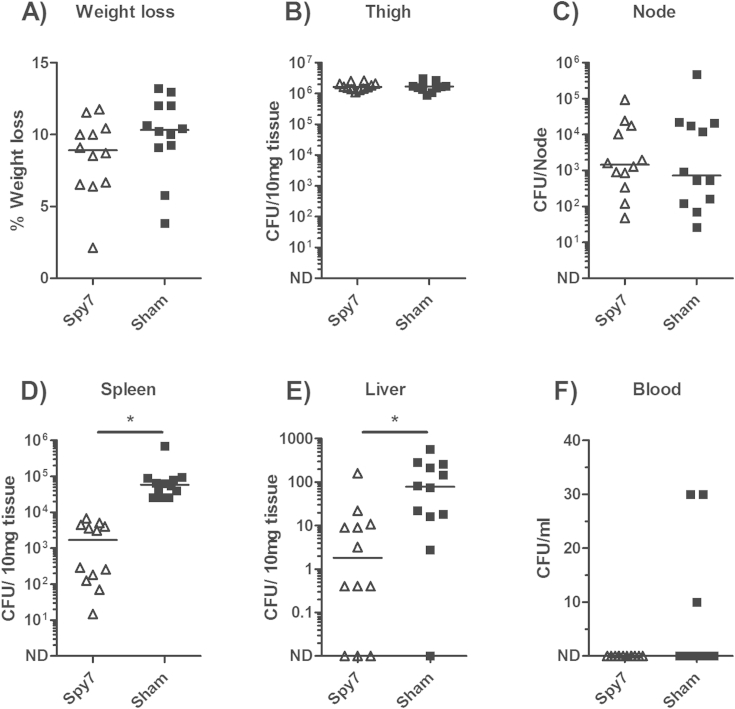
**Spy7 prevents dissemination of M3 *S. pyogenes* from an intramuscular focus of infection**. Two groups of twelve age-matched female FVB/N mice were vaccinated with Spy7 (open triangles) or sham vaccination with PBS and adjuvant alone (closed squares) and challenged with ∼1 × 10^7^ CFU/mouse of M3 *S. pyogenes* (strain H330). Solid lines indicate the median CFU recovered from each organ 24 h post infection. *p < 0.05 two-tailed Mann–Whitney *U*. ND: Not Detected.

The ability of Spy7 to provide protection against other *S. pyogenes* serotypes was then assessed using a representative strain from three other contemporary serotypes of *S. pyogenes* currently circulating in Europe and North America (M3, M12 and M89). In contrast to the previous experiment, no difference in percentage weight loss was observed between the Spy7 vaccinated and sham vaccinated animals infected with serotype M3 *S. pyogenes* (Fig. 3A). The bacterial burden in the infected thigh and draining lymph node remained comparable between groups (Fig. 3B and C). Spy7-induced immunity limited bacterial dissemination to the spleen (Fig. 3D) and liver (Fig. 3E) but had no discernible effect on bacterial burden in the blood (Fig. 3F). Very limited systemic dissemination occurred following intramuscular infection with M12 and M89 *S. pyogenes*, resulting in very low bacterial counts at each organ site and thus making it difficult to adequately compare bacterial spread (Supplementary Fig. 2).

### Impact of vaccination on cytokine and chemokine responses

**Figure 4:**
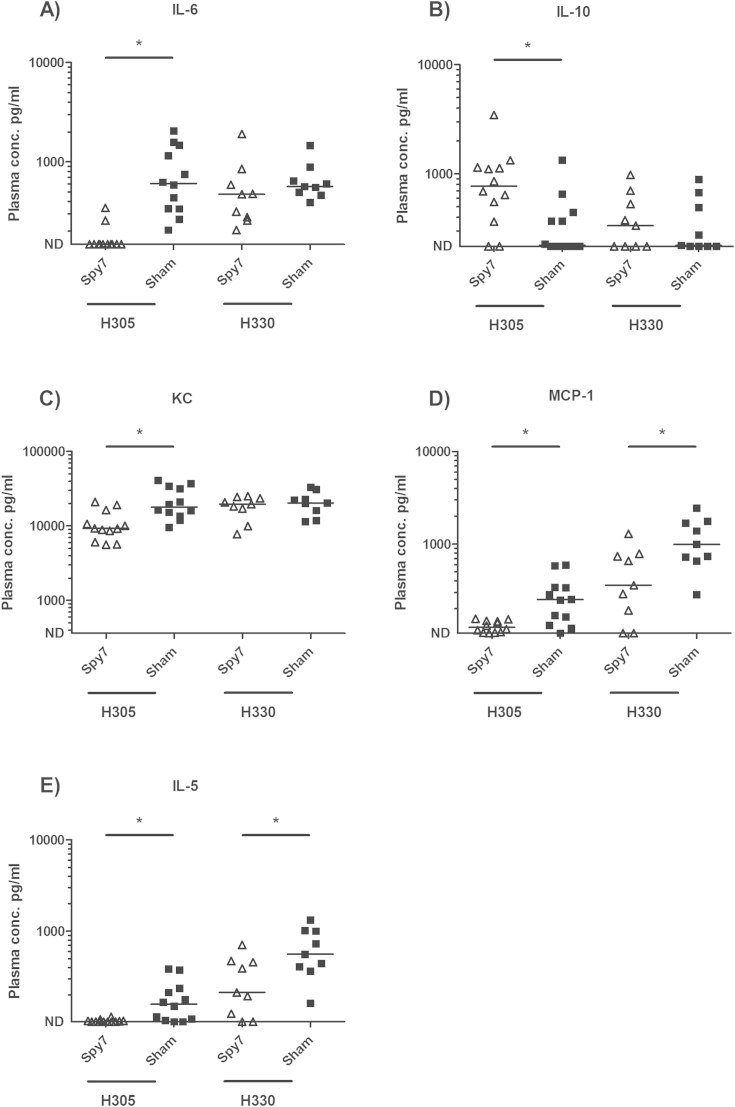
**Vaccination with Spy7 reduces systemic inflammation during experimental *S. pyogenes* infection**. Blood was recovered from the infected animals by cardiac puncture 24 h post infection and the cytokine concentration in plasma was quantified by multiplex analysis. Solid lines indicate the median concentration of each cytokine present within the Spy7 (open triangles) or Sham (closed squares) vaccinated animals. M1 (H305), *n* = 12; M3 (H330), *n* = 9. *p < 0.05 two-tailed Mann–Whitney *U*. ND: Not Detected.

Having established that Spy7 was capable of limiting systemic dissemination in an M1 and M3 infection model, plasma cytokine profiles of the Spy7- and sham-vaccinated groups were compared 24 h post-infection challenge to determine the effect of vaccination on proinflammatory cytokine production in response to infection (Fig. 4). Consistent with changes in weight loss, vaccination with Spy7 reduced levels of IL-6, a recognized marker of severity, in mice infected with M1 *S. pyogenes*, though not those infected with M3 *S. pyogenes* (Fig. 4A). Unlike IL-6, levels of the immunomodulatory cytokine IL-10 were higher in the Spy7-vaccinated M1 group although the significance of this is unclear at present (Fig. 4B). Vaccination also led to a reduction in a number of inflammatory chemokines and cytokines involved in leucocyte chemoattraction and activation (KC and MCP-1) though this was not a universal finding (Fig. 4C and D). This included a clear impact on IL-5 in both M1- and M3-infected mice (Fig. 4E), a cytokine previously linked to superantigen production in experimental *S. pyogenes* infection.^17^ The proinflammatory cytokine TNF-alpha was undetectable in all mice, consistent with previous data from this model.^18^ Unexpectedly, when comparing mice infected with M1 or M3 *S. pyogenes*, levels of IFN-γ, IP-10 and MIG in M3-infected mice exceeded levels measured in M1-infected mice by an order of magnitude, despite bacteraemia being most frequently detected in M1-infected mice (Supplementary Table 2).

### Vaccination with Spy7 induces T cell responses

**Figure 5:**
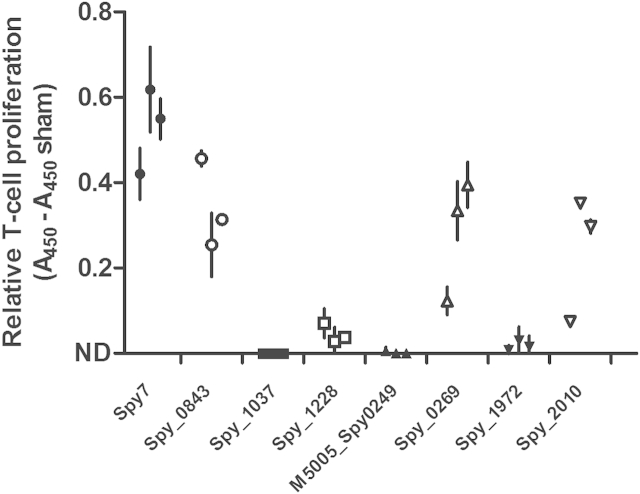
**Vaccination with Spy7 induces a T cell response to Spy7 and its components**. The proliferative response of T cells from the draining inguinal lymph node of mice vaccinated intramuscularly with Spy7 was measured following re-stimulation with Spy7 or the individual vaccine antigens. Symbols differentiate different antigens. Data from individual mice are displayed as mean ± SD of triplicate absorbance readings corrected for non-specific reactivity by subtraction of the mean absorbance generated by cells from a single sham-vaccinated mouse.

To determine if Spy7 vaccination can induce T cell responses, a small cohort of mice was vaccinated with a single dose of Spy7, and cells from the draining lymph node were re-stimulated *ex vivo* with Spy7, or the individual Spy7 antigens. Although from only a small number of mice, the data indicated that Spy7 vaccination induced T cell responses to C5a peptidase (Spy_2010), SpyAD (Spy_0269), cell surface protein (Spy_0843) and, to a more limited extent, nucleoside-binding protein (Spy_1228) (Fig. 5). Together these data suggest that the humoural immune response stimulated by several of the Spy7 antigens may be accompanied by a cognate T cell response.

## Discussion

The human blood product IVIG provides an intriguing surrogate for population-based immunity to *S. pyogenes*. Here we attempted to emulate the protection afforded by experimental IVIG administration using a combination vaccine incorporating seven of the most highly conserved and invariant antigens recognized by the reagent.^12^ Our data demonstrate that Spy7 stimulates a strong humoural immune response *in vivo*, and provides demonstrable protection against two of the most common contemporary *S. pyogenes* serotypes (M1 and M3). While inadequate dissemination of two additional serotypes (M12 and M89) prevented reliable appraisal of the efficacy of Spy7, the available data suggests that the vaccine may indeed provide some protection against these serotypes *in vivo*.

Historically, attempts to develop an *S. pyogenes* vaccine have revolved around the use of the M protein, a multifaceted virulence factor that stimulates type specific immunity during natural infection.^19^ Although the existence of over 100 distinct serotypes initially impeded the development of an effective M protein vaccine, recent analyses have demonstrated that the antigenic diversity of different M protein isoforms may be less extensive than was once thought,^20^, ^21^ and several approaches to M protein-based vaccine design have been evaluated.^4^, ^22^, ^23^ In addition to the M protein, several other antigens have been tested in isolation as vaccine candidates, including six of those included in this study (Table 2). Of these, C5a peptidase, cell surface protein, and SpyAD showed protective efficacy,^5^, ^11^, ^13^ while oligopeptide-binding protein, putative pullulanase, and hypothetical membrane associated protein were not protective.^10^, ^11^ Although immunization with C5a peptidase has been shown to reduce experimental nasopharyngeal colonization, data from invasive *S. pyogenes* infection models yield conflicting results.^5^, ^7^, ^11^ Notwithstanding the success of some single antigen formulations, the risk of vaccine escape mutants and/or clones that do not express the target antigen arising within the vaccinated human population provides a rationale for the combination of several targets to promote consistent clearance of highly pathogenic organisms such as *S. pyogenes*.^10^, ^11^ Indeed the availability of *S. pyogenes* genome data has given rise to a series of systems-based approaches to vaccine design, which have resulted in the development of several combination vaccines.^7^, ^10^, ^11^ Although few of the antigens described herein are reported to protect against *S. pyogenes* infection in isolation, this does not discount their usefulness in novel formulations where the synergistic effects of many targets may contribute to the overall efficacy of the vaccine, inducing neutralizing and opsonic activities. This is particularly true of formulations such as Spy7, which includes proteins involved in complement escape (C5a peptidase),^5^ adhesion (SpyAD and putative pullulanase)^24^, ^25^ and lipoproteins presumed to facilitate substrate binding and nutrient acquisition (maltose/maltodextrin-binding protein, oligopeptide-binding protein and nucleoside-binding protein).

Vaccination with Spy7 reduced disease severity in mice infected with M1 *S. pyogenes*, as evidenced by reduced IL-6 levels, though this effect could not be demonstrated in mice infected with M3 *S. pyogenes*. Disease severity overall may have been greater in M1-infected mice, which were bacteraemic at the 24 h time point, and we speculate that this may have augmented any effect of vaccination on cytokines such as IL-6. Vaccination also influenced the levels of a number of leucocyte chemoattractants such as the IL-8 homologue KC, MCP-1, and IL-5 however, any impact on these cytokines and chemokines may reflect the reduction in bacterial counts observed in the vaccinated groups compared with controls. Of particular note was the effect of vaccination on serum levels of IL-5. IL-5 has previously been shown to be elevated in models of *S. pyogenes* infection potentially in response to superantigens,^17^ although a link with severity and bacterial burden has not been recognized.

Clearly Spy7 would require further refinement and appraisal prior to development of the vaccine, including evaluation of alternative adjuvants that are better suited for use in human vaccination, and evaluation in nasopharyngeal infection. Notably, the methodology employed to identify the Spy7 components did not permit identification of non-proteinaceous antigens such as the group A carbohydrate that may enhance vaccine efficacy and/or coverage.^26^, ^27^ While inclusion of all seven antigens was considered the simplest preliminary approach to development of a multicomponent vaccine, recombinant production and purification of such a large number of molecules may present a significant challenge. Although each component of Spy7 was shown to stimulate humoural immunity; both upon experimental administration and, in most cases, during natural *S. pyogenes* infection, only three (C5a peptidase, SpyAD and cell surface protein) could be shown to generate a cognate T cell response, and were capable of inducing appreciable T cell proliferation upon re-stimulation of lymph node cells from Spy7 vaccinated animals. Interestingly two of these components (C5a peptidase and SpyAD) also reacted strongly with serum from a normal adult, suggesting that the antibody response to these proteins may be long lived. Based on these preliminary findings, it may be appropriate to further refine the components of Spy7 prior to development of the vaccine. Alternatively, it may be possible to produce a chimeric molecule that includes the major antigenic domains of all seven proteins which may further reduce the risk of vaccine escape mutants evolving, by increasing the number of spontaneous mutations required to facilitate immune evasion. Importantly, murine Spy7 antiserum demonstrated no discernible reactivity with human heart valve tissue using an ELISA based assay, suggesting an absence of cross reactive epitopes within any of the selected antigens (data not shown).

Several previous studies have attempted to characterize the *S. pyogenes* surface proteome, and/or the antigens that are recognised by human serum, yielding a large number of potential vaccine candidates.^9^, ^10^, ^28^, ^29^ The proteins identified as potential vaccine candidates in such studies are however influenced by the method employed for screening of target antigens, which have included non-native protein preparations, peptide epitopes, and/or sera of unknown protective activity in previous studies. The current study was unique insofar as the IVIG preparation used to identify potential vaccine candidates was known to be highly protective both *in vitro* and *in vivo*. The use of a highly protective preparation of IVIG as an initial screening technique provided a highly efficient route to vaccine antigen identification in contrast to other studies where large scale *in vivo* experiments are employed to screen candidates.^10^, ^11^

Selection of the subunits for vaccination in the current study was systematic and objective and, unlike previous studies, was not influenced by the novelty of the identified antigens, or previous data relating to their efficacy. Whether inclusion of antigens with previously-demonstrated protective action is of particular importance for the functionality of Spy7 remains unclear. The study enabled rapid identification of potential vaccine candidates without preliminary evaluation in animal models, using an approach that could be extended to other common pathogens. While individual components of Spy7 have previously been evaluated as vaccine antigens, the current study provides much needed rationale for inclusion of these in future combination vaccines.

## Conflict of interest

The authors declare no conflict of interest.

## Figures and Tables

**Table 1:** The isolates used in this study.

Strain	M-type	CovRS status	Disease association
H305	M1	CovS mutant^a^	Scarlet fever
H330	M3	CovS mutant^b^	Puerperal sepsis and toxic shock
H529	M12	WT^c^	Toxic shock
H293	M89	WT^c^	Necrotizing fasciitis

a Stop at residue 318.

b Pro to Ser at residue 285.

c High SpeB producers.

**Table 2:** Selection of Spy7 subunits. Spy7 subunits are displayed in bold.

Gene/ORF	Protein product	Prior reference^d^	Efficacy reported *in vivo*
*scpA*, *M5005_Spy1715*, *Spy_2010*	**C5a peptidase**	5, 7, 11	Carriage: Increased clearance Invasive: Model dependent
*dnaK*, *M5005_Spy1498*, *Spy_1760*	Chaperone protein DnaK^a^	n/a	n/a
*malE*, *M5005_Spy1058*, *Spy_1294*	Maltose/maltodextrin-binding protein^b^	n/a	n/a
*oppA*, *M5005_Spy0249*	**Oligopeptide-binding protein**	11	No
*M5005_Spy0137*	Nucleoside-binding protein^c^	n/a	n/a
*pulA*, *SPy_1972*	**Putative pullulanase**	10	No
*M5005_Spy0942*, *Spy_1228*	**Nucleoside-binding protein**^c^	n/a	n/a
*M5005_Spy0762*, *Spy_1037*	**Hypothetical membrane associated protein**	11	No
*M5005_Spy0651*, *Spy_0843*	**Cell surface protein**	13	Invasive: Increased Survival
*prgA*, *M5005_Spy0229*, *Spy_0269*	**SpyAD**	11	Invasive: Increased Survival

a Chaperone protein DnaK was excluded from Spy7 owing to the cytoplasmic location of the protein.

b Maltose/maltodextrin-binding protein was cloned but could not be expressed recombinantly.

c Nucleoside-binding lipoproteins *Spy_1228* and *M5005_Spy0137* shared a significant degree of amino acid identity, thus only one of these targets (*Spy_1228*) was included in Spy7.

d Prior references are given for antigens previously assessed in active, *in vivo* vaccination experiments only.
