# Supplementary material for: Development of a multicomponent vaccine for Streptococcus pyogenes based on the antigenic targets of IVIG
Source: J Infect. 2016 Apr;72(4):450–9. doi: 10.1016/j.jinf.2016.02.002 (PMC4796040; doi:10.1016/j.jinf.2016.02.002)
Supplement: Supplementary file 1 [file mmc1.docx]

**Supplementary Figure**


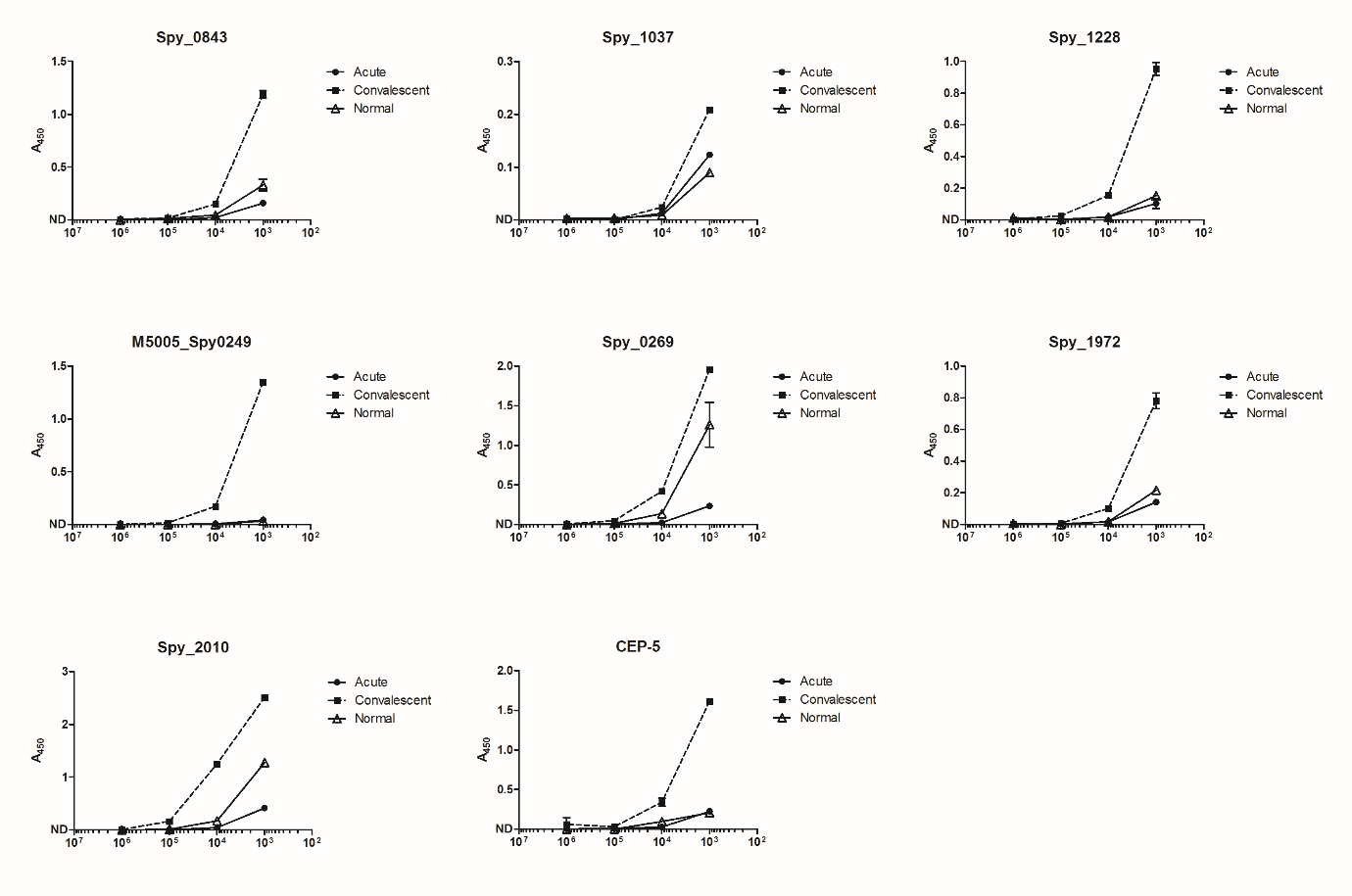


**Supplementary Figure 1. Antibodies to Spy7 antigens develop after natural *S. pyogenes* infection.** ELISA plates were coated with 1 µg/ well of recombinant protein and probed with 10-fold dilutions (1:1000 – 1:1,000,000) of serum from a patient recovered from M1 *S. pyogenes* bacteremia (at presentation and 8 weeks later). Bound antibodies were detected using a 1:2000 dilution of HRP-conjugated goat anti-human IgG. Serum from a healthy adult (normal human serum) was included as a control.

**
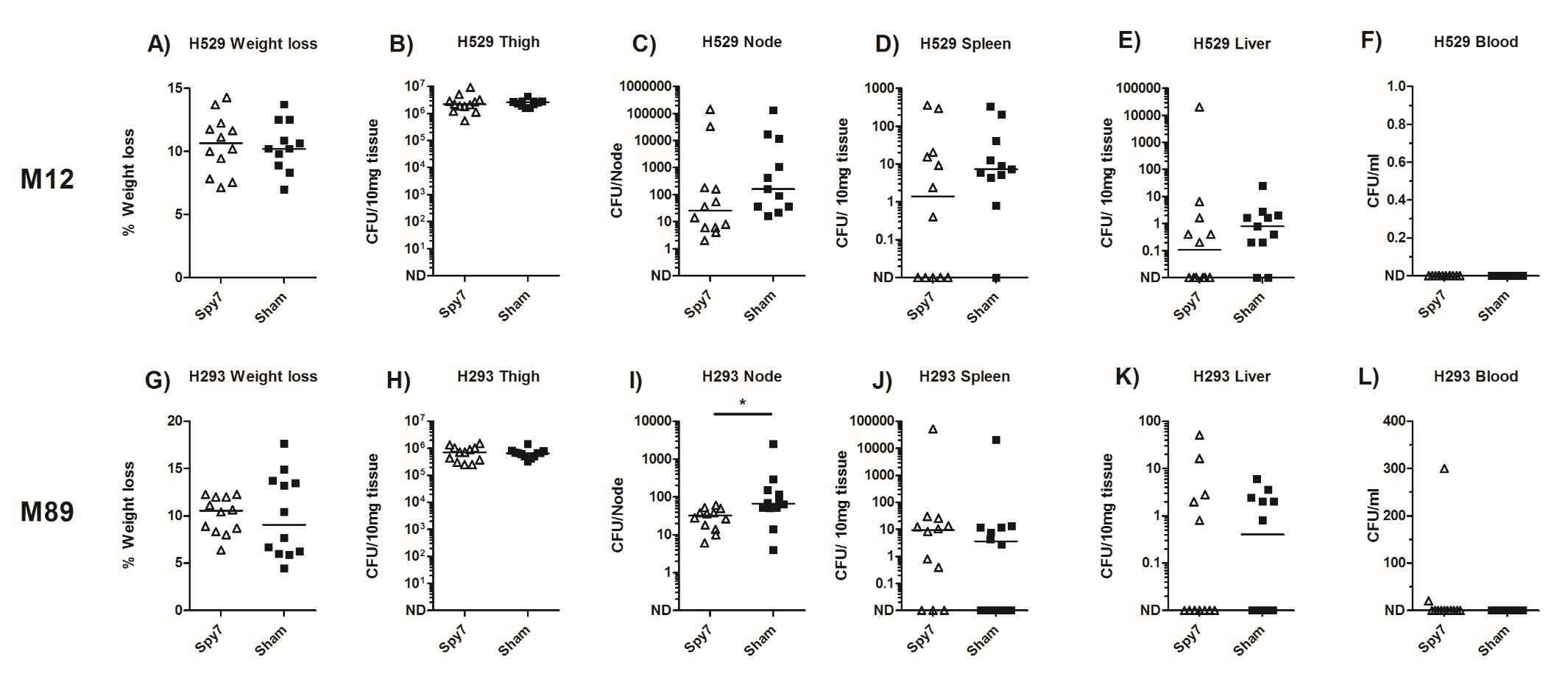
**

**Supplementary Figure 2: The effect of Spy7 administration on M12 and M89 *S. pyogenes* infection.** Two groups of twelve age-matched female FVB/N mice were vaccinated with Spy7 (open triangles) or sham vaccination with PBS and adjuvant alone (closed squares) and challenged with ~1x10^7^ CFU/mouse of M12 or M89 *S. pyogenes*. Solid lines indicate the median CFU recovered from each organ 24 h post infection. *p<0.05 two-tailed Mann-Whitney U. ND: Not Detected.

**Supplementary Table 1: Primer sequences for pET-19b cloning.**

| **Target** | **Primer** | **Sequence** |
| --- | --- | --- |
| Cell surface protein | CSP-F  CSP-R | CCGGATCCAGAAACTTCTGGCCCTGTTGATGATACAG  CCGGATCCTTGCAGAGTGTCGTCCTCTATTCGT |
| Hypothetical membrane associated protein | HMAP-F  HMAP-R | CCGGATCCAAGTCCGATTGAAACCTATACGCATAG  CCGGATCCTTTATTTTTTGGTGGTTGTCAACTGAACG |
| Maltose/maltodextrin-binding protein | MalE-F  MalE-R | CCGGATCCAATGGGTTTGTCATGTATTTCG  CCGGATCCTTTATTTTGTTGCAGCGATATC |
| Nucleoside binding protein | NBP-F  NBP-R | CCGGATCCAGTTGCAATGGTTACCGATACTGGTGGTG  CCGGATCCTCTATTTTTCAGGAACTTTAATGTCACC |
| Oligopeptide binding protein | OppA-F  OppA-R | CCGGATCCAGGAACGACTGATGTGATAACACAAATG  CCGGATCCTTTATTTTTCAACGTGATCAGCTAACTTCTC |
| SpyAD | SpyAD-F  SpyAD-R | CCGGATCCAGGAGAAACGAAGGCGAGTAATACTCACG  CCGGATCCTTCACTTAGATTCCTTACGGAACCTAAATCC |
| Putative pullulanase | PulA-F  PulA-R | CCCATATGGATGGCAATGCTAAATCTGGTGATGG  CCCATATGCGGTAGCAATTGAACGTGGGTAAC |
| C5a peptidase | ScpA-F  ScpA-R  ScpA_(SDM)_-F  ScpA_(SDM)_-R | CCGGATCCAACCAAAACCCCACAAACTC  CCGGATCCTAGAGTGGCCCTCCAATAGC  TTTGCTATCATAGATTGGTTTTTATAAGGCTGACAGATTGCCAAAAT  ATTTTGGCAATCTGTCAGCCTTATAAAAACCAATCTATGATAGCAAA |

**Supplementary Table 2: Impact of vaccination on cytokine and chemokine responses.** Data are displayed as median and range (in brackets) and are given in pg/ml. P values were calculated by two-tailed Mann-Whitney U. ND: Not Detected. N/A: Not Applicable. *p<0.05 (displayed in bold).

|  |  | **M1 (H305)** |  |  | **M3 (H330)** |  |
| --- | --- | --- | --- | --- | --- | --- |
| **Cytokine (limit of detection)** | **Spy7** | **Sham** | **P value** | **Spy7** | **Sham** | **P value** |
| FGF (209.7 pg/ml) | 475.04 (ND - 764) | 362.405 (ND - 743.86) | 0.4317 | 504.88 (ND - 1299.66) | 431.97 (ND - 955.37) | 0.992 |
| GMCSF (94.7 pg/ml) | ND (ND - ND) | ND (ND - 498.11) | 0.3593 | ND (ND - ND) | ND (ND - ND) | N/A |
| IFN-g (80.7 pg/ml) | **ND (ND - 422.3)** | **ND (ND - ND)** | **0.0367 (*)** | **670.02 (ND - 1838.02)** | **1235.97 (732.34 - 3664.8)** | **0.0244 (*)** |
| IL-1a (131.05 pg/ml) | ND (ND - ND) | ND (ND - 368.01) | **0.0165 (*)** | ND (ND - 187.61) | ND (ND - 505.45) | 0.9517 |
| IL-1b (118.4 pg/ml) | ND (ND - ND) | ND (ND - 308.72) | 0.1662 | ND (ND - 154.8) | 142.7 (ND - 308.72) | 0.1086 |
| IL-2 (34.8 pg/ml) | 65.05 (49.3 - 69.77) | 65.465 (50.87 - 217.48) | 0.7290 | 80.1 (58.52 - 87.79) | 87.03 (67.73 - 116.07) | 0.1903 |
| IL-4 (224.9 pg/ml) | ND (ND - 251.91) | ND (ND - ND) | 0.3593 | ND (ND - ND) | ND (ND - 266.29) | 0.3741 |
| IL-5 (101.5 pg/ml) | ND (ND - 115.12) | 158.32 (ND - 384.36) | **0.0025 (*)** | **211.9 (ND - 706.54)** | **556.91 (161.79 - 1324.53)** | **0.0422 (*)** |
| IL-6 (147.3 pg/ml) | **ND (ND - 343.57)** | **604.895 (205.41 - 2055.82)** | **<0.0001 (*)** | 470.24 (205.41 - 1904.69) | 563.62 (389.8 - 1458.29) | 0.1903 |
| IL-10 (216.7 pg/ml) | **771.885 (ND - 3454.61)** | **221.94 (ND - 1339.62)** | **0.0235 (*)** | 335.04 (ND - 975.83) | 221.04 (ND - 886.64) | 0.7816 |
| IL-12 (39.35 pg/ml) | 114.07 (77.37 - 154.43) | 117.625 (62.83 - 226.56) | 0.6232 | 310.66 (56.39 - 513.92) | 347.89 (154.15 - 824.28) | 0.2581 |
| IL-13 (126.7 pg/ml) | ND (ND - 309.88) | ND (ND - ND) | 0.1662 | ND (ND - ND) | ND (ND - ND) | N/A |
| IL-17 (35.75 pg/ml) | ND (ND - ND) | ND (ND - ND) | N/A | ND (ND - ND) | ND (ND - ND) | N/A |
| IP-10 (99.75 pg/ml) | **102.965 (ND - 359.79)** | **ND (ND - 169.95)** | **0.0284 (*)** | 778.52 (ND - 1874.4) | 1317.88 (630.14 - 4761.5) | 0.0631 |
| KC (362.1 pg/ml) | **9237.155 (5583.48 - 20968.78)** | **18000.275 (9609.36 - 41145.42)** | **0.0029 (*)** | 19625.48 (7770.77 - 25166.87) | 20330.82 (11466.39 - 33220.8) | 0.6665 |
| MCP-1 (108.85 pg/ml) | **125.745 (ND - 155.99)** | **250.98 (ND - 592.85)** | **0.0047 (*)** | **358.55 (ND - 1291.26)** | **995.89 (284.25 - 2450.78)** | **0.0340 (*)** |
| MIG (43.85 pg/ml) | 498.24 (ND - 2349.41) | 538.795 (122.33 - 1340.62) | 0.4187 | 8483.65 (139.39 - 23379.58) | 5205.15 (1195.46 - 26599.21) | 0.8633 |
| MIP-1a (190.8 pg/ml) | ND (ND - ND) | ND (ND - 721.92) | 0.3593 | ND (ND - ND) | ND (ND - ND) | N/A |
| TNF-a (115.25 pg/ml) | ND (ND - ND) | ND (ND - ND) | N/A | ND (ND - ND) | ND (ND - 130.55) | 0.1693 |
| VEGF (28.85 pg/ml) | ND (ND - 267.32) | ND (ND - 114.68) | 0.5726 | ND (ND - 85.02) | ND (ND - 49.94) | 0.5468 |
